# A novel wearable device to deliver unconstrained, unpredictable slip perturbations during gait

**DOI:** 10.1186/s12984-019-0602-0

**Published:** 2019-10-17

**Authors:** Corbin M. Rasmussen, Nathaniel H. Hunt

**Affiliations:** Department of Biomechanics, University of Nebraska at Omaha, 6160 University Drive South, Omaha, NE 68182 USA

**Keywords:** Slips, Falls, Balance, Task-specific Training, Gait, Perturbations, Wearable Technology

## Abstract

**Background:**

Task-specific perturbation training is a widely studied means of fall prevention, utilizing techniques that induce slips or slip-like perturbations during gait. Though effective, these methods only simulate narrow ranges within the larger space of possible slipping conditions encountered in daily life. Here we describe and test a novel, wearable apparatus designed to address these limitations and simulate a diverse range of slipping disturbances.

**Methods:**

The device consists of wireless triggering and detachable outsole components that provide adequate friction with the floor when secured to the wearer’s foot, but suddenly create a low-friction surface underfoot upon release. “Benchtop” tests were carried out to quantify device triggering characteristics (i.e. cutting temperature, release delay) and the resulting friction reduction. The device was also tested on six healthy young adults (3 female, age 23 ± 2.4 years), who walked with and without the device to observe how gait kinematics and spatiotemporal parameters were influenced, then performed 12 walking trials ending with a slip delivered by the device. Each participant also completed a survey to obtain opinions on device safety, device comfort, slip realism, and slip difficulty. A linear mixed effects analysis was employed to compare subject spatiotemporal parameters with and without the apparatus, as well as correlation coefficients and root mean square errors (RMSE) to assess the impact of the device on lower limb gait kinematics. Slip onset phases, distances, directions, velocities, and recovery step locations were also calculated.

**Results:**

This device rapidly diminishes available friction from static coefficients of 0.48 to 0.07, albeit after a substantial delay (0.482 ± 0.181 s) between signal reception and outsole release. Strong correlations (*R* > 0.93) and small RMSE between gait kinematics with and without the device indicate minimal effects on natural gait patterns, however some spatiotemporal parameters were significantly impacted. A diverse range of slip perturbations and recovery steps were successfully elicited by the device.

**Conclusions:**

Our results highlight the efficacy and utility of a wearable slipping device to deliver diverse slip conditions. Such an apparatus enables the study of unconstrained slips administered across the gait cycle, as well as during different locomotor behaviors like turning, negotiating slopes, and level changes.

## Background

Task-specific training has gained popularity in rehabilitation as an effective intervention to regain [1, 2] and reinforce [3] motor skills. This methodology closely mimics the sensorimotor and environmental interactions of the target task, thereby forcing the patient or subject to repetitively execute the movements necessary to accomplish the goal of the task [4]. This paradigm is widely utilized in studies of gait stability and fall prevention by administering repeated perturbations to an individual in the form of simulated trips and slips [5–7].

Many successful task-specific techniques and apparatuses have been reported in the literature to study slipping perturbations in particular, the cause of 22–25% of falls in the community [8, 9]. Of these, the most widely used are sudden, transient accelerations on a treadmill during otherwise steady-state walking [10–14], one or more unlockable sliding platforms embedded in a level walkway [15–17], and oiled areas encountered along a path [18, 19]. Important advances in our understanding of reactions to “slip-and-fall” events across age groups, as well as supporting evidence for the potency of perturbation training on stability, have stemmed from the use of these perturbation methods. For example, arm swing in the sagittal plane mitigates deviations in trunk posture following a slip, an action that older adults are less able to utilize [19]. Individuals appear to transition from a reactive to anticipatory mechanism during perturbation training, characterized by an anteriorly-shifted center of mass (CoM), altered lower limb kinematics, and reduced heel contact velocity [17, 20]. Increases in compensatory step lengths [14], improved measures of stability immediately after slip onset and recovery step touchdown, and spatial-temporal adaptations [11] have also been reported. The adjustments obtained from treadmill slip training in particular can be scaled to match the intensity of perturbation [14], and those from sliding platforms can be generalized to other tasks of varying context similarity [21–23]. While these results have only been shown for their respective perturbation methods to date, the findings illustrate the potential plasticity of learned slip recovery strategies. After participating in a perturbation protocol, subjects have retained their acquired stabilization skills for months [22], and quickly readapt after periods over a year [23, 24].

Clearly, available methods for administering task-specific slip perturbations are capable of eliciting valuable insight on slip attributes, response movements, and stability adaptations. However, they have practical limitations associated with producing slips that are unexpected [6, 25], kinematically unconstrained [26], and that represent the variety of slip conditions encountered in daily life. Foot velocity, sliding distance, slip location, and/or slip direction are restricted with these procedures, narrowing the range of unique disturbances that can be applied. Specifically, emulated perturbations from sliding platforms and treadmills are limited entirely to the anterior-posterior direction, while actual slips possess a notable mediolateral component [26]. Slippery floor surfaces provide the most realistic disturbances when encountered, but they suffer from a high degree of predictability with repeated exposure [6, 25]. These constraints are inherent to the devices and methods used to administer the perturbations.

In order to reduce or remove these restrictions and allow for more realistic slips to be imposed, a new type of apparatus is required. Our aim was to develop such a device. Namely, this apparatus would deliver repeated slipping perturbations that were unconstrained in direction and magnitude, yet be spatially and temporally unpredictable to the wearer and controlled by an attending researcher or clinician. In addition, the slips delivered by this device could be delivered at any point during stance phase. We elected to pursue a wearable design, as this would remove the spatial predictability limitation while still allowing slips to be delivered during over-ground walking. Indeed, a previous exploration of the design space for variable-friction devices concluded that, compared to various environment-based approaches, a wearable, shoe-based option was the most promising [27]. Following development of the initial prototype, subject and non-subject tests were performed to quantify the functional attributes of the device, as well as assess and validate its ability to deliver diverse perturbations and elicit recovery reactions. Based on the results of these tests, we outline improvements to be made for future device iterations. Finally, we also highlight a number of previously inaccessible gait modalities and research topics that are unlocked with the use of a wearable slipping device.

## Materials and Methods

### Device Design

**Fig. 1:**
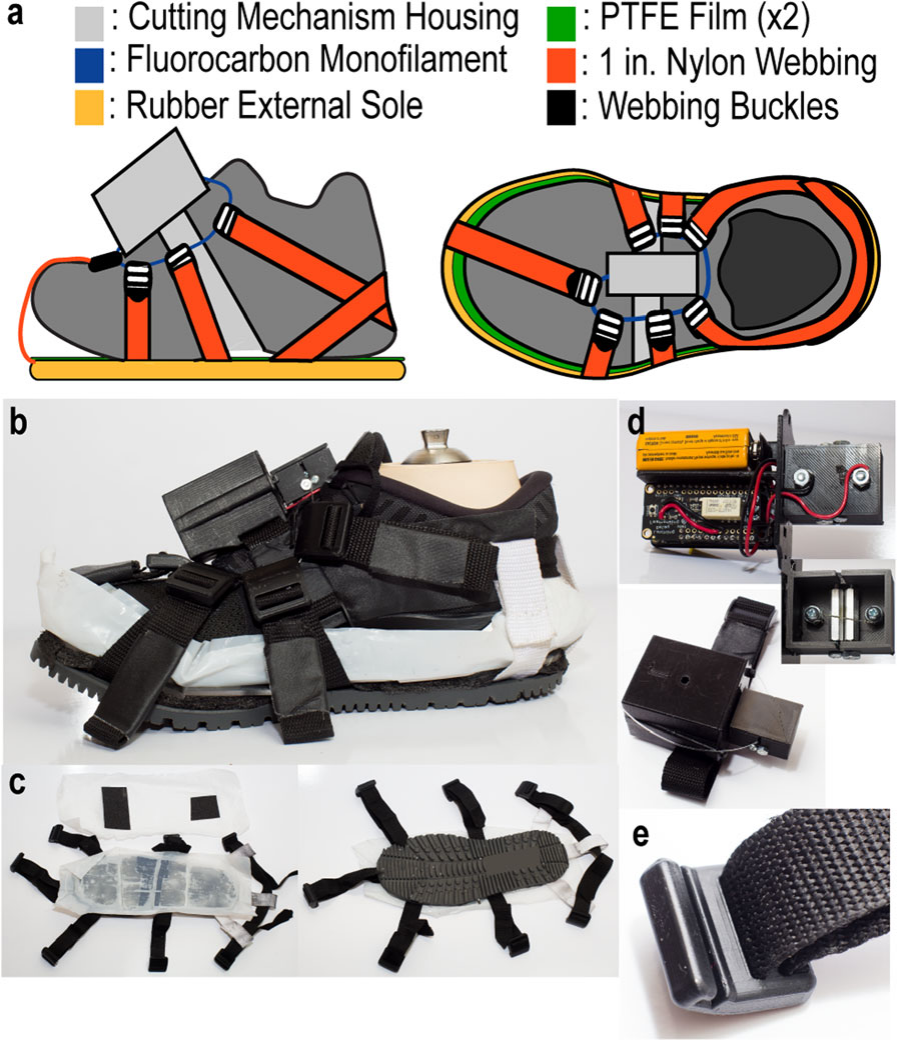
**a** Color-coded diagram of WASP, individually highlighting the components. **b** The entire WASP assembly fitted to a shod prosthetic foot. **c** Superior and inferior views of the WASP outsole with PTFE sheets and straps. **d** The trigger mechanism, detailing the internal electronics and the nichrome interface. **e** An example of the custom 3D printed buckles used to connect the two components via the fluorocarbon filament

Our device (Fig. 1a, b), which hereafter will be called the Wearable Apparatus for Slipping Perturbations (WASP), consists of two interacting components: a detachable outsole (Fig. 1c) and a wirelessly controlled release mechanism (Fig. 1d). The key element of the detachable outsole is a modified rubber boot sole with seven, one-inch wide nylon webbing straps fastened around the perimeter with screws. The free ends of these straps are threaded through custom 3D printed buckles (Fig. 1e) that enable the attachment of the outsole to the release mechanism. Adhered to the top of the boot sole is a layer of foam to cover the screw heads and provide a flat surface, followed by two thin sheets of polytetrafluoroethylene (PTFE) film. PTFE is a low-friction compound colloquially known as Teflon™. A 1:1 mixture of water and personal lubricant [28] is applied between the PTFE sheets. Two pieces of slip-resistant safety tape were attached to the dorsal surface of the top PTFE layer to provide friction with the wearer’s shoe, which in turn only allows sliding to occur between the lubricated PTFE layers.

The release mechanism, contained entirely in a 3D printed housing, is affixed to the wearer’s shod foot via nylon strap and buckle. Within the housing is a Bluetooth-enabled relay that, when activated through a smartphone application (Adafruit Industries; New York City, NY), supplies power from a 9-V battery to a length of six 38 AWG nichrome wires twisted together (Fig. 1d). Nichrome is a high-resistance alloy of nickel and chromium, characterized by its ability to dissipate large amounts of energy as heat yet maintain a resistance to oxidation. This nichrome strand is housed in its own case apart from the battery and electronics. A 13.5-in. circumference loop of 0.028-in. thick fluorocarbon filament is passed through the nichrome case, perpendicular to and in contact with the wire. This filament loop provides the attachment point for the detachable outsole component via the 3D printed buckles (Fig. 1e). The entire apparatus is secured tightly to the wearer’s foot by pulling the excess nylon webbing through these buckles.

In its attached state, WASP is intended to facilitate natural walking by providing adequate friction between the floor and plantar surface of the rubber outsole. Upon reception of a wireless trigger, the relay channels electricity to the nichrome strand, rapidly heating it. The fluorocarbon filament loop in contact with the nichrome is severed by the intense heat, which relieves the tension on the loop from the 3D printed buckles and releases the detachable outsole [see Additional file 1]. Once released, the wearer is exposed to the low-friction interface between the lubricated PTFE sheets, ultimately causing a slip perturbation [see Additional file 2]. In order to deliver repeated slips, the outsole must be prepared and reattached to the wearer’s foot between trials. Additional lubricant is applied between the two PTFE film layers to replace what was lost during the previous trial and to ensure a low friction surface after the next outsole release. To reattach the outsole, the severed fluorocarbon filament loop is removed and a new loop is placed in the housing across the nichrome strand. The buckles of the outsole are then hooked onto the new loop and the nylon straps tightened in the same way as described before.

### Functional Testing

We performed a number of benchtop analyses to quantify the key functional characteristics at play while using WASP. To assess the temperatures realized by the nichrome wire through repeated activation cycles, we recorded the wire with an infrared thermal imaging camera (FLIR T540, FLIR Systems, Inc.; Wilsonville, OR) at 30 Hz through 20 consecutive cycles. Each trial was separated by a “rest period” between seven and ten min in length to closely mimic the protocol used in our human data collections described later. The camera was calibrated to an emissivity of 0.65 [29], positioned 0.3 m away from the cutter box, and set to record within a temperature range between 150° and 3000° Celsius. This experiment began with a new battery, and its voltage was recorded immediately before each cycle to examine battery drain with repeated use.

Because WASP relies on the rapid heating of a nichrome strand to melt a fluorocarbon filament, there is an inherent delay between reception of the triggering signal and the instant of release. To determine the length of this delay, we recorded 20 cutting trials with a high-speed camera (Fastec Imaging Corp.; San Diego, CA) recording at 1000 Hz. WASP was affixed to a shod prosthetic foot as in Fig. 1b and equipped with an LED programmed to illuminate when the triggering signal was received. The number of frames between the lighting of the LED and the first observable movement of the filament were counted and converted to the corresponding amount of time. As in the previously described temperature tests, seven to ten min separated each trial. Also, a new battery was used and its charge measured prior to each cut.

**Fig. 2:**
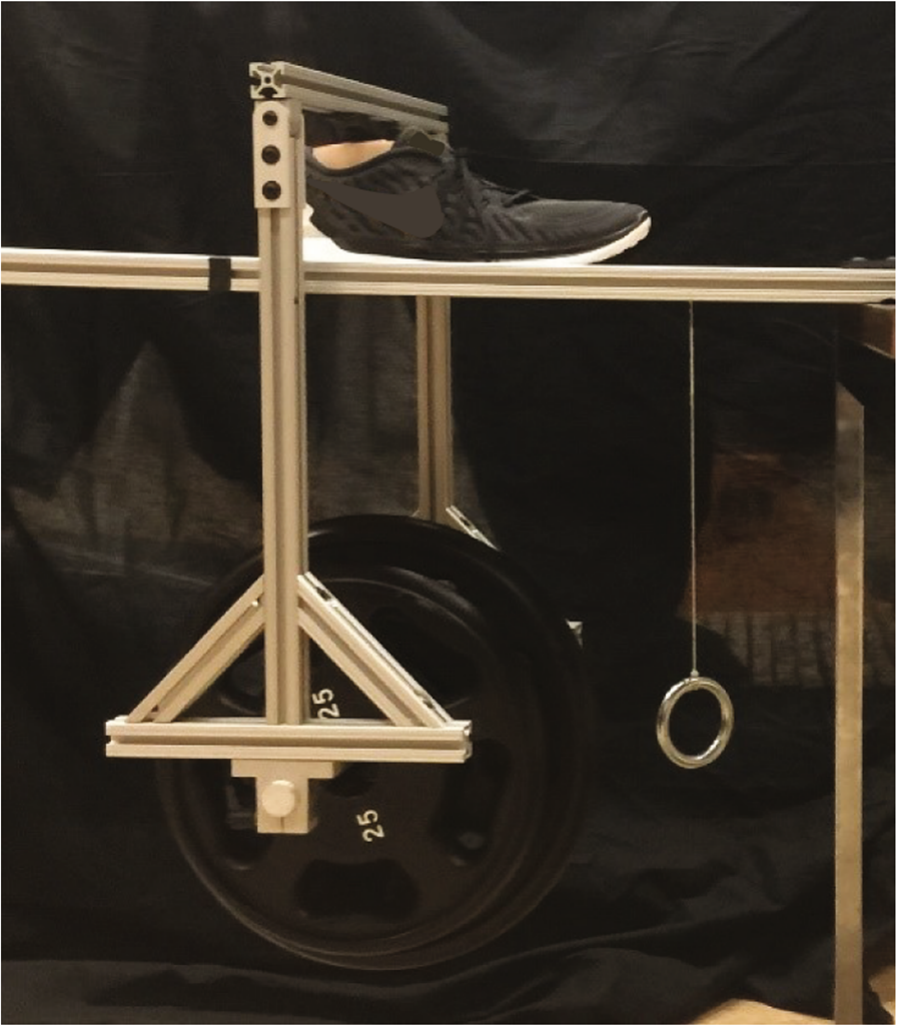
Static friction testing setup used to obtain SCoF values, recorded from a similar perspective as seen in this photo with a video camera

Slipping is facilitated by inadequate friction between an individual’s foot and the supporting surface they are standing or walking on. To imitate this mechanism, it is essential for WASP to instantly transition from providing sufficient friction for walking to creating a low-friction surface underfoot. To discern the magnitude of this change, a ramp and weight carrying rig (Fig. 2) were assembled to test the static coefficients of friction (SCoF) of the three interfaces that may be encountered while using WASP: WASP-floor, unlubricated PTFE-PTFE, and PTFE-PTFE lubricated with the 1:1 mixture [28]. To simulate the lab floor, a spare flooring tile was used as the sliding surface for the WASP-floor condition. Weights of 75 lbs., 180 lbs., and 240 lbs. were tested in all conditions except WASP-floor to analyze the effect of varying body weights on the supplied friction (240 lbs. was not performed in the WASP-floor condition due to concerns about device safety). Each weight condition was performed ten times. Trials consisted of slowly elevating one end of the ramp to create a slope. A camcorder (Canon Inc.; Tokyo, Japan) documented each trial at 30 Hz. An image was extracted from each video at the instant that the rig began to slide down the ramp. Ramp angles relative to a pendulum were measured using ImageJ [30] and used to derive the angle between the ramp and horizontal. The tangent of this angle was taken as the SCoF for each trial.

### Study Participants

Approval for the following protocol was obtained from the University of Nebraska Medical Center Institutional Review Board, and all procedures were in accordance with pertinent regulations and guidelines. We piloted our device on six healthy, young adults (mean ± SD {range} age: 23 ± 2.4 {20–27} yrs., height: 1.76 ± 0.08 {1.71–1.91} m, mass: 73.33 ± 13.96 {56.25–90.27} kg, 3 females), who were all screened and consented to participate in this study. Exclusionary criteria included cardiopulmonary, musculoskeletal, and neurological conditions or injuries that may influence normal gait patterns. Anthropometric measurements (i.e. body weight, height, limb dominance) were collected from each participant.

### Experimental Protocol

Subjects were outfitted with a form-fitting compression suit, lab-provided athletic shoes, and a full-body retroreflective marker set. A safety harness was also fitted to each subject, which was connected to a fall arresting system mounted to a rail on the ceiling. A load cell rated for 500 kg (HT Sensor Technology Co. LTD; Xi’an, China) was placed in series with the system to measure the forces exerted on the harness by each subject during slipping trials. If the measured force supported by the harness exceeded 30% of the subject’s body weight, the trial was classified as a fall [31].

To obtain a description of each participant’s natural walking kinematics, we asked them to walk across an eight meter walkway at a self-selected comfortable speed without wearing WASP. Marker trajectories were collected at 120 Hz by a 17-camera motion capture system (Motion Analysis Corp.; Santa Rosa, CA). To ensure that any observed biomechanical differences between walking with and without WASP could be attributed to the device, all participants were connected to the ceiling-mounted safety harness during these trials. Gait speed was observed in real time using an electronic timing system (Dashr, LLC; Lincoln, NE). Following this, subjects were given a five-minute rest period, during which WASP was attached to their dominant foot. A WASP outsole was affixed to the non-dominant foot of each subject as well to prevent differences in limb length.

**Fig. 3:**
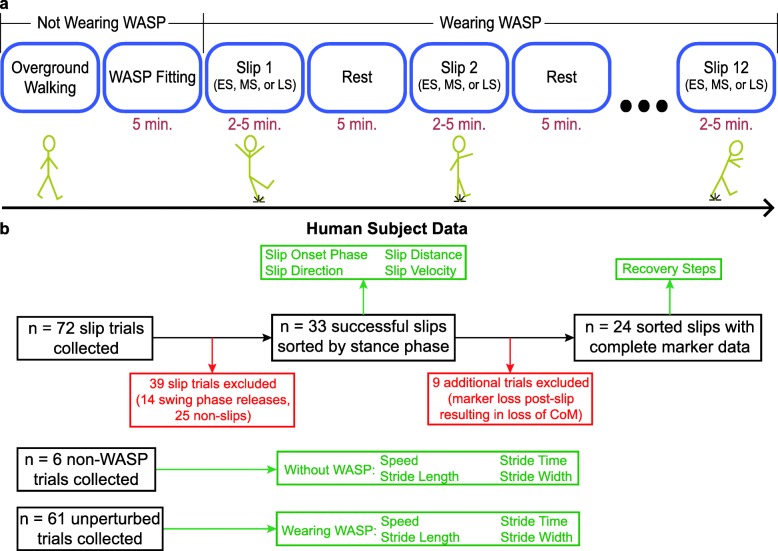
**a** An illustration of the protocol used during human subject data collections. **b** An illustration of the human subject trials included and excluded in data analysis

While wearing WASP, each participant was asked to walk back and forth across the walkway at the same self-selected comfortable speed as in the previous non-WASP condition. Feedback was provided to the subjects during the trials when necessary to keep them as close to that speed as possible. All trials were between two and five min in length, before which subjects were informed that they may or may not experience a slip [15]. Following this time, WASP was triggered by an attending researcher in an attempt to coincide with either heel strike, mid-stance, or toe-push. Because of the delay between signal reception and detachment, the researcher was required to anticipate the desired gait phase. Trial duration and slipping phase were randomized using a Matlab script (Mathworks Inc.; Natick, MA). After triggering, subjects were allowed a five-minute rest period while WASP was reset. This protocol was repeated 12 times for each subject (Fig. 3a), after which a post-study questionnaire was completed to extract their perceptions of device comfort, slip realism, recovery difficulty, and safety while wearing WASP.

### Data and Statistical Analysis

Gait events were defined using the coordinate-based algorithm described by Zeni and colleagues [32]. Spatiotemporal gait parameters (i.e. stride time, stride length, stride width, and gait speed) were then calculated for unperturbed walking both with and without WASP. To assess the effect of the wearable device on natural gait patterns, a linear mixed effects analysis was used to determine the effect of wearing WASP on gait speed (*n* = 6 without WASP, 61 with WASP), stride time, stride length, and stride width (*n* = 42 without WASP, 394 with WASP). The presence of WASP in a given trial was entered into the model as a fixed effect, while subjects were treated as a random effect to resolve non-independencies within the fixed effect (i.e. presence of WASP). The alpha level for these analyses was set at 0.05. In addition, root mean square error (RMSE) and correlation coefficients were calculated for lower limb kinematics observed while walking with and without WASP to quantify the impact of the device.

Slip onset times were determined visually by three individuals using the model videos built using Visual3D (C-Motion Inc.; Germantown, MD). Each individual labeled the frame in the video they believed corresponded with the instant the slip began. Discrepancies between individuals were resolved in the following ways: 1) the frame identified by the majority was used, or 2) if none of the individuals were in agreement, then the midpoint between the two closest frames was used. If individuals could not distinguish a slip at any point in a trial, the trial was excluded from further analysis (*n* = 25, Fig. 3b). Due to the uncontrolled delay of the release mechanism, 14 trials ended with the WASP outsole releasing during swing phase of the target limb and therefore did not cause a perturbation. These trials were also excluded from further analysis. The remaining 33 trials were sorted into early stance (ES), mid-stance (MS), and late stance (LS) categories based on their time of occurrence during stance. ES was defined as 0–33.3%, MS as 33.4–66.7%, and LS as 66.8–100% of stance phase. Slip distances, directions, and velocities were obtained from the motion of the sliding foot’s CoM. Distance was taken as the difference between the positions of the foot CoM at slip onset and at the following toe-off, while velocity was the maximum attained during the same period. Direction was calculated relative to the subject’s bearing. The location of the next step following slip onset, which we will call the “recovery step”, was derived from the X and Y coordinates of the unperturbed foot’s CoM relative to those of the whole-body CoM. Due to missing whole-body CoM position data at the instant of recovery step touchdown, nine of the remaining 33 trials were not included in the recovery step calculations (Fig. 3b).

## Results

### Functional Characteristics

**Fig. 4:**
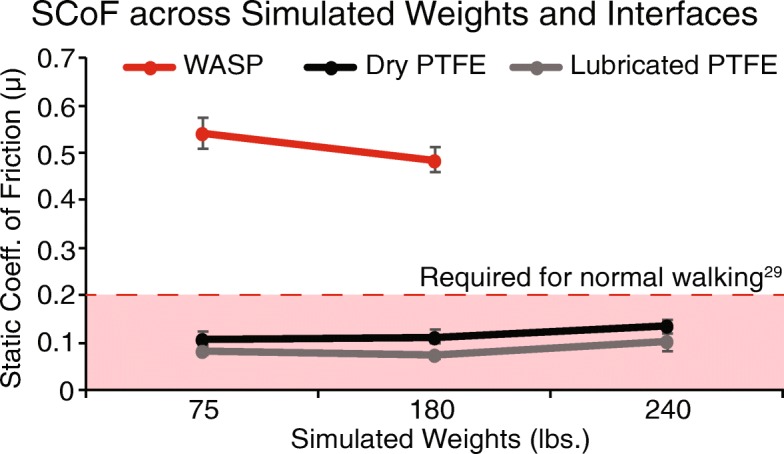
Average SCoF coefficients (±1 SD) at 75, 180, and 240 lbs.

The average SCoF observed between the WASP outsole and tile floor surface was 0.54 ± 0.03 with a 75 lbs. load and 0.49 ± 0.03 under a 180 lbs. load. Friction is severely reduced following activation regardless of lubrication state, with dry and lubricated coefficients of 0.11 ± 0.01 and 0.08 ± 0.01 at 75 lbs., 0.11 ± 0.01 and 0.07 ± 0.004 at 180 lbs., and 0.13 ± 0.02 and 0.10 ± 0.02 at 240 lbs. (Fig. 4). Following reception of the trigger signal, the average (± SD {range}) delay until outsole release was 0.482 ± 0.181 {0.311–0.871} seconds. This delay was due to the time required for the nichrome wire to reach a temperature sufficient to sever the monofilament. The maximum temperature attained by the nichrome element (576.6 ± 29.7 {528.5–640.1}°C) consistently occurred after outsole release at one second post-triggering, as this was the instant that the mechanism’s relay ceased to supply power. The maximum temperature steadily dropped with repeated use. Available voltage to the system from the onboard 9-V battery decreased at a logarithmic rate through multiple uses, likely explaining the general downward trend in peak temperature.

### Walking With and Without WASP

**Fig. 5:**
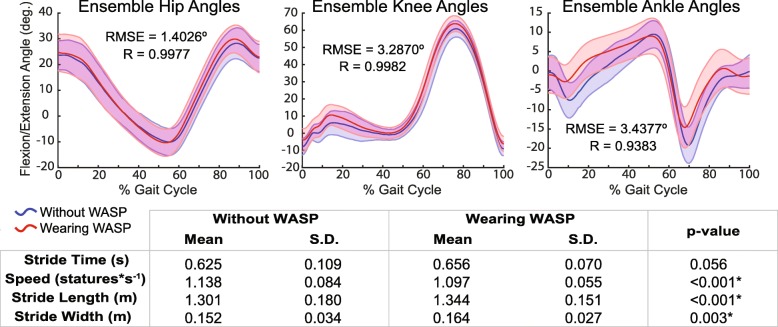
Sagittal plane lower extremity kinematics and spatiotemporal gait parameters observed during over-ground walking both with and without WASP

Wearer perceptions of their gait pattern were mixed, with 50% of responses indicating that a natural gait was maintained. Respondents generally felt safe from losing their balance while wearing the device (8.0 ± 1.8 {5–10}), but were less approving of its comfort (5.7 ± 1.7 {3–9}). Stride times while wearing WASP were not significantly different than without (0.656 ± 0.070 s with WASP vs. 0.625 ± 0.109 s without WASP; t (65) = 1.94, *p* = 0.056). However, the differences in gait speed (1.097 ± 0.055 statures/s with WASP vs. 1.138 ± 0.084 statures/s without WASP; t (434) = − 7.09, *p* < 0.001), stride length (1.344 ± 0.151 m with WASP vs. 1.301 ± 0.180 m without WASP; t (434) = 3.37, *p* < 0.001), and stride width (0.164 ± 0.027 m with WASP vs. 0.152 ± 0.034 m without WASP; t (434) = 2.95, *p* = 0.003) were all significant (Fig. 5).

Wearing WASP did not appear to substantially impact the kinematics of the lower extremities during over-ground walking, as indicated by low RMSE values (RMSE = 1.4026°, 3.2870°, and 3.4377°, respectively; Fig. 5) and very strong correlations between hip, knee, and ankle angles measured both with and without the devices (R = 0.9977, 0.9982, and 0.9383, respectively; Fig. 5). While wearing WASP, subjects on average exhibited an additional 0.3° of maximum hip extension and 1.7° of maximum hip flexion, as well as an additional 4.5° of knee flexion during loading and 3.0° during mid-swing. The ankle was the most effected, with a reduction of 4.8° in plantarflexion immediately after heel contact, of 0.5° in dorsiflexion at terminal stance, and of 4.6° in plantarflexion at toe-off.

### Administered Perturbations

**Fig. 6:**
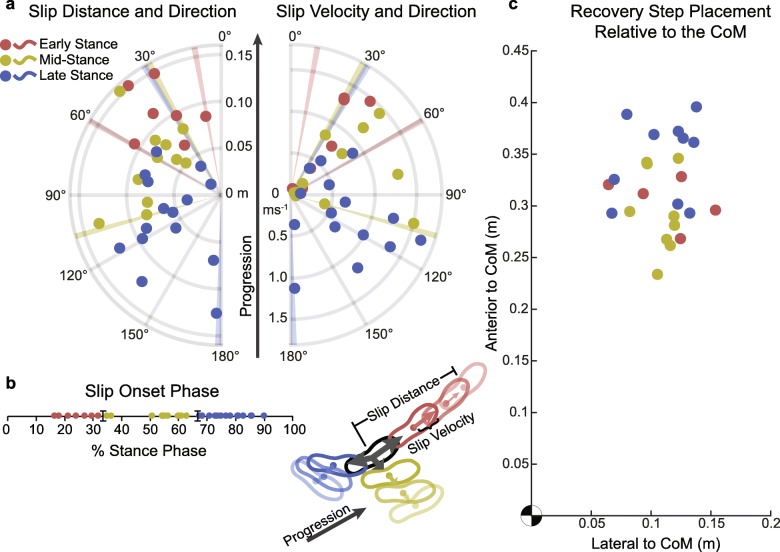
**a** Attributes of administered slips coded by slip phase. **b** The distribution of administered slips across stance phase. **c** Recovery step locations relative to the location of the whole-body CoM

None of the successfully administered perturbations met the criteria to be classified as a fall. Successfully inflicted slips were broadly distributed, occurring between 16 and 91% of stance phase (Fig. 6b). Slipping directions relative to the direction of progression fell between 10° and 178°, with angles generally growing as slips occurred later in stance (Fig. 6a). Slip distances ranged from a minimum of 1.6 cm to a maximum of 15.4 cm, and peak velocities from 4.5–163.2 cm/s. Recovery steps also covered a broad range of positions that varied in both the anterior-posterior and medial-lateral directions (Fig. 6c). In contrast to the observed slipping angles, phase-distance, phase-velocity, and phase-recovery step relationships are not immediately clear. Participants reported that the perceived severity of the slips was low (2.8 ± 1.7 {1–6}), but were more moderate when asked if they seemed realistic (5.3 ± 1.5 {3–7}).

## Discussion

Through the experiments described above, we assessed the functional characteristics of our novel slipping device and its utility to deliver unrestricted slipping perturbations in a laboratory setting. Our results demonstrate WASP’s ability to do this at any point during stance phase, which was a primary intention of our device. This is achieved through rapid reduction of friction underfoot when triggered, thereby perturbing the base of support (BoS) of the wearer. Administered disturbances were highly variable, and appeared to be influenced at least in direction by onset phase. In its attached state, the device had a minimal effect on the wearers’ lower limb gait kinematics and caused small yet significant changes to a number of spatiotemporal parameters.

**Fig. 7:**
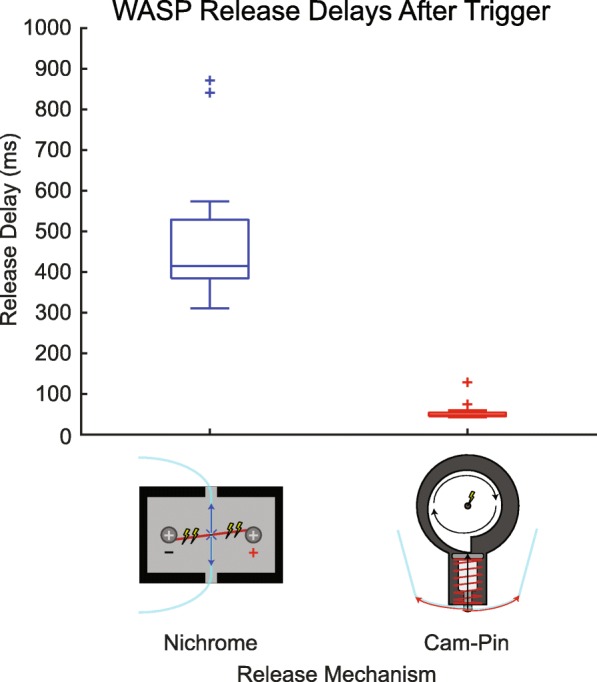
A comparison of release delays following reception of the trigger command using the nichrome method employed in this study and an alternative cam and follower mechanism developed later. As reported, the mean ± 1 SD delay for the nichrome mechanism was 0.482 ± 0.181 s, while the cam and follower delay was 0.056 ± 0.02 s


Also through our observations, we identified a number of improvements to be addressed in future iterations of wearable slip perturbation devices. First and perhaps the most important of these is the uncontrollable variability in outsole release, which renders the precise targeting of specific phases of gait by the experimenter difficult. This stems from two particular aspects of our device. The first aspect is the inconsistent delay between reception of the trigger command and outsole release. Because this release is caused by rapid melting of the monofilament, the delay is dependent on factors not easily controlled with our current mechanism. To address this issue, we developed an alternative release mechanism that uses a cam and follower system to detach the outsole after performing the experiments outlined previously. This mechanism still receives a wireless trigger signal from the experimenter, but instead of supplying power to a strand of nichrome wire, the Bluetooth-enabled relay delivers electricity from a battery to a small DC motor. This motor is outfitted with a 1000:1 gear ratio transmission in order to produce a high degree of torque. The driveshaft of the motor connects to a drop cam, which depresses a spring-loaded pin that protrudes from the housing when in contact with the peak of the cam (see illustration in Fig. 7, Additional file 3). To attach the outsole, a loop of monofilament is threaded through the buckles of the nylon webbing straps and hooked to the protruding pin of the trigger mechanism. When activated, the motor turns the cam, which allows the spring loaded pin to rapidly retract into the housing. This retraction releases the monofilament loops and thereby the outsole from the foot [see Additional file 3]. Reattaching the outsole requires the cam and pin to be reset to their “armed” positions by turning the motor, followed by hooking the monofilament back onto the protruding pin. Because outsole release does not rely on severing a loop of filament, there is no need to replace the filament after every trial. Using the same release delay testing method performed on the nichrome mechanism, we found that the cam and follower mechanism is both faster and more consistent (0.056 ± 0.02 s, Fig. 7). Based on this result, we plan to utilize the cam and follower release component in future studies using this wearable slip device, as improved control over the instant that friction is reduced will allow more targeted, consistent perturbations to be administered. Second, because the current form of WASP relies on an experimenter to manually release the outsole through a Bluetooth-connected device, one must be very accurate and consistent to target specific gait phases or events repeatedly. Nevertheless, there will undoubtedly be errors between the intended and actual times of release. This potential for error can be remediated by developing an automatically released system. Such a system could rely on signals from footswitches or an inertial measurement unit to estimate gait phases and trigger outsole release. This would require the user to simply “arm” the device before a trial to activate at a specified gait phase during the next step, after a number of strides, or after a predetermined length of time. The uncontrollable variability created by the two aspects above should not be confused with the desired “experimenter-controlled” variability in slip onset phase, which is determined primarily by the phase of stance at which the device is activated. If the goal was to deliver all slips at the same phase, a user would simply trigger the device at the same phase for every trial (for the present device, it should be triggered roughly 500 ms before the intended phase of release). Further, while the difference in supplied friction before and after triggering was substantial, slip severity could be increased through greater reduction of traction underfoot. Increased severity would increase the likelihood of eliciting falls, therefore providing greater challenges to the wearer’s balance recovery abilities. The ability to modulate the available friction after release would also be advantageous to actively change the severity of administered slips, analogous to controlling distance or velocity with treadmills or sliding platforms [13]. This feature in a wearable device could prove valuable to a progressive perturbation training program. Finally, from a clinical perspective, our device suffers from the need for a moveable overhead harness in order to be used safely, as well as a relatively large area for the wearer to walk through. These limitations are shared by all over-ground slip perturbation methods, and they may preclude settings that lack such space or equipment from using the device. Despite these limitations, we believe the unconstrained and unpredictable nature of wearable slipping devices may complement the knowledge already gained from existing methods by facilitating the study of unique slip features, which will be described in detail to follow. Successfully addressing our device’s drawbacks through alternative release mechanisms and materials will streamline this research and allow for more targeted, consistent perturbations to be delivered.

**Additional file 3.** Example Cam and Follower Release. This video shows the cam and follower release mechanism developed after the experiments described in this article. This alternative mechanism was built to address the unreliable release delay that was a limitation of the nichrome, and will be used in future experiments with our wearable device.


Our wearable device demonstrated the capability to produce slips with variable onset times. This capability enables the investigation of the role biomechanical context plays in slip severity, specific reactive movements, and fall rates. For example, differences in slip attributes that follow slips initiated at distinct phases may be due to the natural course of kinematic and dynamic context over stance. In the case of ES slips, the anteriorly-directed shear forces may carry the slipping foot forward and away from the rest of the body [33]. Combined with the loss of shear ground reaction forces (GRFs), this creates an exaggerated moment arm that pitches the body backward. Recovering from this backward pitch is more challenging than respective forward rotations [34, 35]. MS and LS slip recoveries may benefit from less compromising body positions and passive dynamics. For MS disturbances specifically, the affected foot appears to slide laterally from the CoM, likely causing a momentary rotation of the body in the opposite direction. The unaffected foot at this time is in swing, and may be able to oppose the motion of the CoM by redirecting its trajectory and planting lateral to the CoM. LS slips might require even less intervention, as the generally posterior-directed slip may set the CoM in motion anteriorly toward the unperturbed foot that is already positioned ahead of the body. Each phase may offer a distinct set of initial conditions from which an individual must find a unique, successful recovery strategy. Although our preliminary results on reactive movements are not sufficient to infer such effects of slip onset phase, they indicate differences that may be detectable in a future study using WASP.

The wearable apparatus delivered slipping perturbations diverse in distance, direction, and velocity in response to a range of onset times. Likewise, diverse slips that occur when navigating the environment depend only on the available friction between the support surface and the outsole as well as the distribution of forces acting over that interface [33], allowing an extremely complex and variable space of possible slip attributes. Compared to previous work, the slips provoked by WASP were slightly shorter and generally slower. In studies where these were controlled, displacements ranged from 1 cm up to 38 cm with velocities between 10 cm/s and 900 cm/s [12–14]. When not constrained, average displacements of 78 cm and 61 cm, as well as peak velocities of 200 cm/s and 184 cm/s, were observed in fallers and non-fallers, respectively [16]. These protocols only produced translations in a single plane [12–14, 16], however, imposing unnatural constraints on the administered perturbations [26]. As suggested by Troy and Grabiner, greater experimental control is gained at the expense of realism of the task [26]. Indeed, context similarity seems to be an important factor to the success of task-specific perturbation training, as transfer of learned stabilization skills from sliding platform training to an over-ground unconstrained slip was significantly greater compared to the same training done on a treadmill [13]. A wearable perturbation device may offer even greater task specificity of lab-induced slips to those encountered in the environment than both methods, therefore future research should investigate the importance of task specificity by comparing improvements in stability gained from training with a wearable device to those from sliding platform and treadmill protocols.

Because slips caused by WASP are highly variable and unconstrained, the compensatory steps and foot orientations in response may have to be equally diverse in order to successfully recover. Recovery steps, seemingly by being placed in the direction a fall would otherwise occur [36], reconfigure the BoS to arrest the destabilizing angular momentum of the body through an opposing angular impulse [37] and maintain body weight support. Multiple studies have aimed to characterize reactive stepping ability [10, 38–40] and report improvements following a perturbation training protocol [12, 14]. It may be possible to supplement these advances through the use of more ecologically valid means, as a broader range of effective recovery steps could be practiced.

A wearable device such as WASP enables the application of perturbation training to other locomotory modes and environments that have not been fully examined in the existing literature, yet describe a significant portion of the steps taken day-to-day. The vast majority of gait perturbation studies have focused on linear, forward gait across a level surface, yet walking in the community is far more complex. Turning comprises approximately 35–45% of all steps taken [41], and a study by Crenshaw and colleagues indicated that 20% of reported falls in their sample occurred while changing direction [9]. The required coefficient of friction needed to safely execute a turn is 0.45 [42], more than twice the value for straight walking [33]. Slopes present a wide range of potential conditions that demand unique biomechanical adaptations to traverse. Walking directly up or down an inclined surface generates significantly larger anterior-posterior shear GRFs [43], requiring greater friction as the magnitude of the incline increases [44]. These forces peak at different times during stance as well [43], which may lead to different “critical phases” where the risk of a slip is greatest. Crossing slopes in any other direction creates asymmetrical functional leg lengths [45], resulting in unique body positions that may influence the repertoire of effective recovery strategies available in the event of a slip. In addition, WASP allows for other mechanical consequences of the perturbation to be examined that are inhibited by the restricted nature of other techniques. One crucial example may be the freedom of foot orientation to change during a slip. Perturbed foot yaw may alter the area of the BoS and therefore the possible locations of the CoP from which to generate countering GRFs. To our knowledge, foot orientation during or after a slip has yet to be investigated.

## Conclusions

Our study indicates that wearable apparatuses like WASP are capable of instigating variable slipping perturbations during stance phase, in turn eliciting unconstrained slip mechanics and recovery reactions. Slips encountered in nature are intrinsically complex due to the limited number of constraining factors present. By closely mimicking this complexity, future work using this device will investigate the possibility of developing a more comprehensive repertoire of reactive stabilization strategies that is effective against a broader range of slipping conditions and environments.

## Supplementary information

**Additional file 1.** WASP Release Example Video. This high-speed video was recorded as part of the data set to characterize the release delay, as described in the Materials and Methods section. The WASP device is attached to a shod prosthetic foot as it would be to a subject, and activated through the reception of a trigger command issued by a Bluetooth-connected device. Upon receiving the command, the nichrome within the green housing heats up rapidly, eventually melting through the monofilament loop that the outsole is attached to. Note that the red LED visible in the video was not present during the human subject trials described within the manuscript, only during the benchtop analyses to indicate when the trigger command was received.

**Additional file 2.** WASP Example Subject Trial. This video shows the WASP device in action while the wearer is walking over-ground, demonstrating how it delivers a slip perturbation. This video was recorded solely for demonstration purposes and was not used for data collection in any way. Further, the individual in the video is an undergraduate member of our research team, and did not participate in the present study as a subject.

## Data Availability

The datasets generated during the current study are available from the corresponding author upon reasonable request.
